# Inhibition of CaMKIV relieves streptozotocin-induced diabetic neuropathic pain through regulation of HMGB1

**DOI:** 10.1186/s12871-016-0191-4

**Published:** 2016-05-23

**Authors:** Xin Zhao, Le Shen, Li Xu, Zhiyao Wang, Chao Ma, Yuguang Huang

**Affiliations:** 1 Department of Anesthesiology, Peking Union Medical College Hospital, CAMS&PUMC, No.1, Wangfujing, DongCheng District, Beijing, 100730 China; 2 Institute of Basic Medical Sciences Chinese Academy of Medical Science, Neuroscience Center, School of Basic Medicine Peking Union Medical College, Department of Anatomy, Histology and Embryology, 100005 Beijing, China

**Keywords:** CaMKIV, Diabetic neuropathic pain, HMGB1, Dorsal root ganglion, Neuron

## Abstract

**Background:**

The pathogenesis of diabetic neuropathic pain is complicated and its underlying mechanisms remain unclear. Calmodulin-dependent protein kinases (CaMKs) IV (CaMKIV), one of CaMKs, regulates several transcription factors in pain mechanisms. High-mobility group box 1 (HMGB1) is a key mediator in diabetic neuropathic pain. This study aims to find the roles and mechanisms of CaMIV in diabetic neuropathic pain.

**Methods:**

Diabetic animal models were constructed by injecting with streptozotocin (STZ) intraperitoneally. They were randomly divided into seven groups (*n* = 6 per group): Naive, Normal Saline, STZ, STZ + Sham, STZ + DMSO and STZ + KN93 (an inhibitor of CaMKIV) (50 μg), STZ + KN93 (100 μg), which received KN93 (50 or 100 μg) intrathecally after the administration of STZ. Phospho-CaMKIV (pCaMKIV) and HMGB1 expression in rat dorsal root ganglion (DRG) and RAW264.7 cell line were measured by western blot. Distribution of pCaMKIV immune reactivity in different subpopulations of DRG neurons was measured by double-immunofluorescence staining.

**Results:**

The pCaMKIV and HMGB1 in DRG significantly increased after STZ administration, and pCaMKIV can regulate the expression of HMGB1 based on both cellular and animal models. Pretreatment with CaMKIV inhibitor attenuated STZ-induced mechanical allodynia and thermal hyperalgesia, as well as reduced HMGB1 expression in the DRG.

**Conclusions:**

This study demonstrates that CaMKIV can relieve STZ-induced diabetic neuropathic pain. The mechanism of this function depended on the process: pCaMKIV localized in the nuclei of DRG neurons and regulated HMGB1 which was an important mediator of neuropathic pain. These findings reported CaMKIV may be a potential target or important node in relieving diabetic neuropathic pain.

## Background

Diabetic neuropathic pain is one of the most common complications of both type 1 and type 2 diabetes. However, information regarding diabetic neuropathy is insufficient to propose an efficient therapy for such chronic pain. To further understand the mechanisms underlying the development of diabetic neuropathy, type 1 and type 2 diabetes animal models have been used to study this phenomenon [1, 2]. Streptozotocin (STZ)-induced type 1 diabetes is a typical model for diabetic neuropathy, because systemically administered STZ exerts a cytotoxic effect on pancreatic β cells [3].

Calmodulin-dependent protein kinases (CaMKs), including CaMKI, CaMKII and CaMKIV, are important mediators of intracellular Ca^2+^ signaling, which perform important roles in cell physiology. These serine-threonine (Ser/Thr) protein kinases are activated upon Ca^2+^/CaM binding [4]. CaMKI and CaMKII are expressed in all mammalian cells [5]. CaMKIV is found predominately in cells of the nervous and immune systems [6]. CaMKIV is activated and translocated into the nucleus upon its phosphorylation by an upstream CaMKs kinase (CaMKK) in the cytoplasm [7]. The nuclear, autonomously active form of CaMKIV phosphorylates numerous proteins involved in transcription regulation [8]. However, the effects of CaMKIV on neuropathic pain remain unclear.

High-mobility group box 1 (HMGB1) is a DNA-binding protein located in the nuclei of most mammalian cells. HMGB1 performs structural and transcriptional activities by binding to chromatin. Moreover, HMGB1 is either actively secreted or can be passively released by injured or necrotic cells [9]. Emerging evidence has shown that HMGB1 is a proinflammatory mediator of chronic pain development, including neuropathic pain [9]. In db/db mice, a model of type 2 diabetes, the development of mechanical allodynia is associated with the upregulation of HMGB1 protein in the spinal cord, and intrathecal injection of the neutralizing antibody against HMGB1 inhibited mechanical allodynia [10]. However, whether CaMKIV is involved in STZ induced neuropathic pain through modulation of spinal HMGB1 in rats remains unclear.

The present study investigated the effects of CaMKIV on diabetic neuropathic pain, as well as the relationship of CaMKIV with HMGB1 expression in dorsal root ganglion (DRG). STZ-induced diabetic models were imployed to investigate the variation of pCaMKIV and HMGB1 in DRG via Western blot (WB) and immunehistochemical (IHC) assays. KN93, an inhibitor of CaMKIV [11], and CaMKIV-siRNA were also used to study the relationship between pCaMKIV and HMGB1. The results indicated that CaMKIV is involved in STZ-induced diabetic neuropathic pain via regulation of HMGB1.

## Methods

### Animals

Male Sprague–Dawley rats (180 g to 200 g) were purchased from the Experimental Animal Center of the Chinese Academy of Medical Sciences. The animals were allowed to adapt to the laboratory for minimus of 2 h prior to testing, and used only once. All animal procedures and experimental protocols in this study were approved by the “Institutional Animal Care and Use Committee (IACUC)” of Chinese Academy of Medical Sciences (“Tab of Animal Experimental Ethical Inspection” number: Acuc-A02–2015–005; IACUC Chairman: Minli Li). All protocols were also consistent with the NIH Guide for Care and Use of Laboratory Animals (NIH Publication No. 80–23).

### Intrathecal catheter implant surgery

Each rat was anesthetized with 50 mg/kg sodium pentobarbital intraperitoneally (i.p.) and an intrathecal catheter was implanted as described by Yaksh and Rudyb [12]. Briefly, a polyethylene tubing (PE-10) filled with sterile normal saline was inserted through a small incision on the atlantooccipital membrane and extended caudally to the lumbar enlargement of the spinal cord. Studies involving rats with chronic intrathecal catheters were conducted after implantation. Rats were monitored daily for signs of neural dysfunction and were removed from the study if neurological dysfunction was noted. Overall, no animals were excluded in the diabetic model experiments.

### Induction and assessment of diabetes in rats

Experimental diabetes was induced by a single i.p. injection of STZ (Sigma-Aldrich Co., St. Louis, MO, USA). The STZ solution was prepared freshly by dissolving normal saline and was injected intraperitoneally at a dose of 65 mg/kg on the seventh day after surgery as previously described [13]. Diabetes induction was assessed by weekly measurement of the tail vein blood glucose level by using a blood glucose meter (HEA-214, OMRON, USA). Body weight was also monitored. Only rats with blood glucose concentration exceeding 240 mg/dL were considered diabetic and used for the study.

### The effect of KN93 on diabetic models

The rats were randomly divided into seven groups (*n* = 6 per group): Group Naive; Group Normal Saline (NS), which received only the NS; Group STZ, which received STZ; Group STZ + Sham, which sham operation after administration of STZ; Group STZ + DMSO, which received DMSO intrathecally after the administration of STZ; Group STZ + KN93 (50 μg), which received KN93 (50 μg) intrathecally after the administration of STZ; Group STZ + KN93 (100 μg), which received KN93 (100 μg) intrathecally after the administration of STZ.

KN93 obtained from Calbiochem was dissolved in sterile DMSO 20 μl and injected intrathecally on 15, 17, 19 days after the STZ administration respectively. The dose of KN93 was chosen based on previous publications [14].

### Behavioral tests

Paw mechanical withdrawal threshold (MWT) was used to measure mechanical allodynia and was assessed by testing the left hind paw withdrawal response to Electronic Von Frey filaments (Stoelting Co., USA). Brisk withdrawal or paw flinching was considered a positive response. Each rat was tested five times per stimulus strength.

Paw thermal withdrawal latency (TWL) was used to measure thermal hyperalgesia and performed by using a heat pain stimulator (PL-200, Taimen Biotech Company, Chengdu). The measurement was repeated five times for each rat (interval ≥ 5 min), and the mean was calculated as PWL for this measurement.

### Western blot analysis

Total protein was extracted from the L4 and L5 DRGs using methods described earlier [15]. The primary antibodies against pCaMKIV, CaMKIV, HMGB1 (1:500; Santa Cruz, USA) were used in Western blot (WB). To verify equal loading of protein, the blots were reprobed with primary monoclonal antibody against β-actin (ProteinTech Company, USA).

### Immunohistochemistry

Immunofluorescent labeling of the following markers was performed on different group rat lumbar DRG cryosections using the methods as previously described [16]. The primary antibodies in the immunohistochemistry: rabbit polyclonal and mouse monoclonal antibodies against pCaMKIV, HMGB1, isolectin B4 (IB4), protein gene product 9.5 (PGP9.5), calcitonin gene-related peptide (CGRP), 1:200, Santa Cruz. The secondary antibodies used: Alexa Fluor 555-conjugated donkey-anti-mouse, 1:500; Alexa Fluor 488-conjugated donkey-anti-rabbit, 1:500, Invitrogen.

The cells were visualized using a laser confocal microscopic imaging system (Nikon A1; Nikon Co.Ltd., Tokyo, Japan) with a water immersion 10 objective lens (Plan Apo 60x1.20 PFS WI) and a 1 st dichroic mirror (405/488/561/640). The green signal was excited by 488 nm light from an Ar laser and red signal was excited by 561 nm light from a DPSS laser. The images were 400 times magnification and the scale bar in Fig. 4 was 20 μm.

### Transfection of siRNA

RAW 264.7 cells (2*10^4^) were plated in 0.5 ml of growth medium (without antibiotics) in each well of a 24-well plate. The sequence of negative control siRNA (N.C. siRNA) and CaMKIV siRNA obtained from Zhang X et al. [11]. The protocol is also comes from Zhang X [11].

### Statistical analysis

Data were presented as means ± standard deviation. A behavioral test was evaluated by two-way repeated measures analysis of variance (ANOVA). The expression of pCaMKIV and HMGB1 were analyzed by one-way or two-way ANOVA with Student Newman Keuls post hoc analysis. *P*-values of <0.05 were considered statistically significant.

## Result

### Validation of diabetic models

**Fig. 1:**
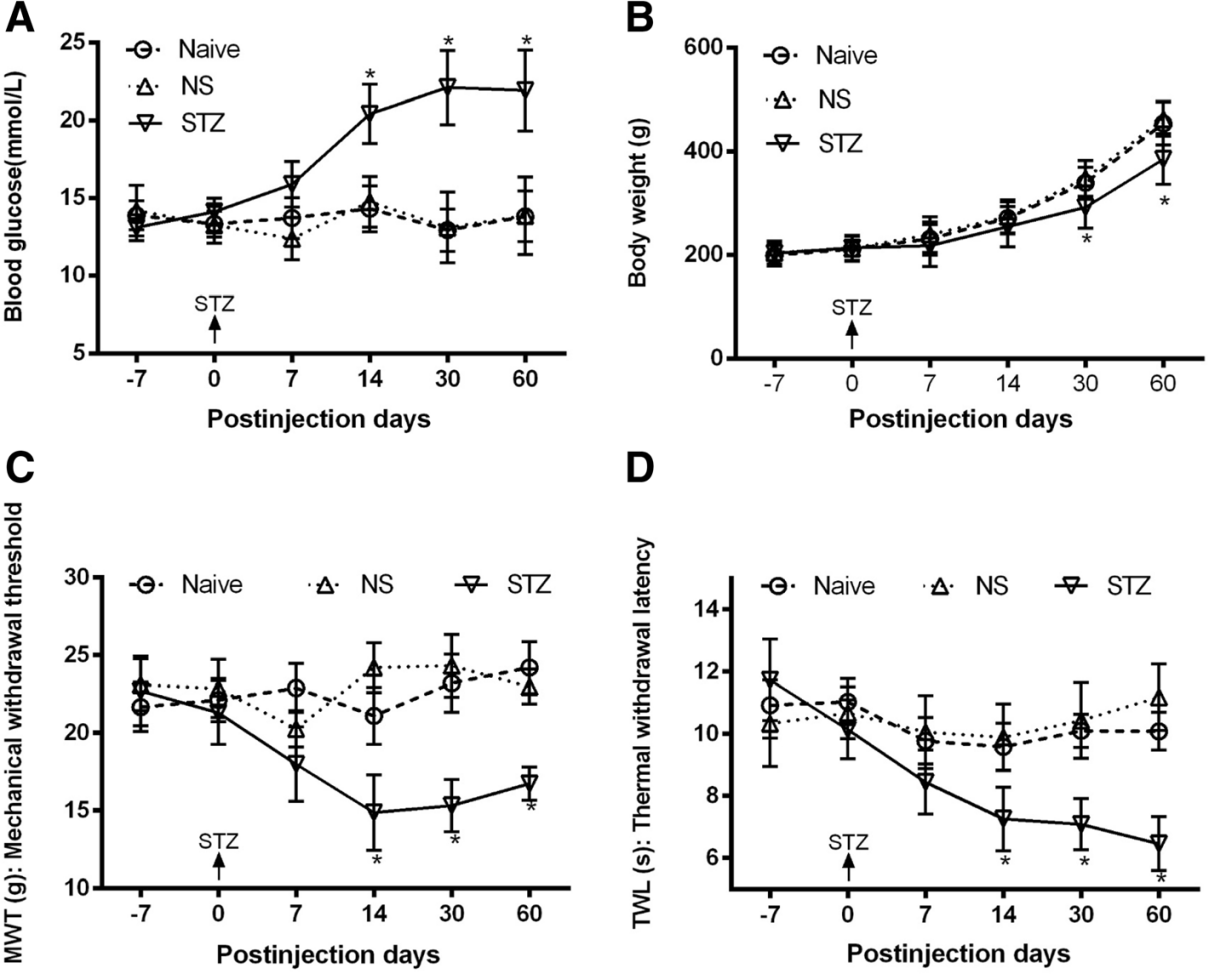
Changes in blood glucose level, body weight, MWT, and TWL in STZ-induced diabetic rats. **a**. Diabetes induction was assessed by measurement of blood glucose level by using a blood glucose meter. **b**. Changes in body weight in control and diabetic rats. **c**. STZ-induced mechanical allodynia. **d**. STZ-induced thermal hyperalgesia. The data are presented as means ± SD. Naive, group naive; NS, group NS, which received only the NS; Group STZ, which received STZ; *P < 0.05 compared with the Naive and NS groups, *n* = 6 per group

At 14 days after STZ injection, the blood glucose level of the STZ group was significantly higher than those of the Naive and NS groups (Fig. 1a) (*P* < 0.05). STZ-injected rats exhibited a significant reduction of weight gain relative to those of Naive and NS groups after 1 month (Fig. 1b) (*P* < 0.05). To confirm damage to the nervous system, MWT was measured, and a significant decrease in MWT was found in diabetic rats after 14 days of STZ injection as compared with the Naive and NS groups (Fig. 1c) (*P* < 0.05). Marked TWL was also observed in diabetic animals, as evidenced by a reduction in pain thresholds, compared with the Naive and NS groups after 14 days (Fig. 1d) (*P* < 0.05). These results suggest that the STZ-induced diabetic model can be used in this research.

### pCaMKIV expression was significantly increased in diabetic models

**Fig. 2:**
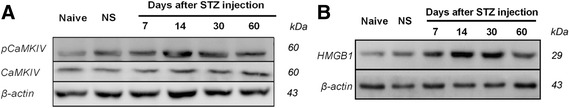
Changes in pCaMKIV and HMGB1 expression in DRG of STZ-induced diabetic rats, as detected by Western blot. **a**. Upregulation of pCaMKIV, but not CaMKIV after STZ injection. **b**. Upregulation of HMGB1 after STZ injection. Data are presented as means ± SD. *P < 0.05 compared with the Naive and NS groups, *n* = 6 per group

The pCaMKIV levels in DRG were investigated by Western blot and showed a significant increase in diabetic models, whereas the CaMKIV expression level did not exhibit considerable change. Gray scanning displayed a marked change in pCaMKIV levels after STZ treatment (Fig. 2a). The level of HMGB1, which is an important regulator of neuropathic pain, increased after STZ treatment, and this finding occurred simultaneously with pCaMKIV (Fig. 2b).

### pCaMKIV regulates HMGB1 expression

**Fig. 3:**
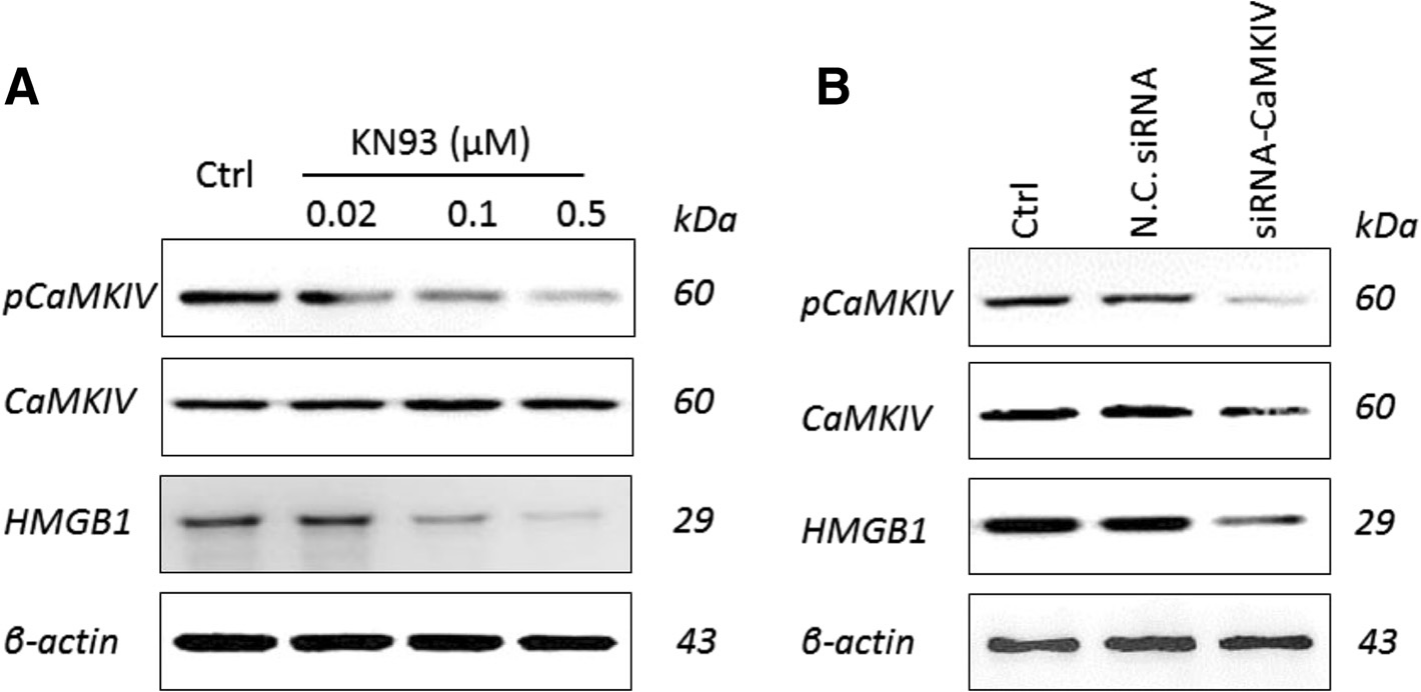
pCaMKIV can regulate HMGB1 expression. **a**. Effects of KN93 on the expression of pCaMKIV, CaMKIV and HMGB1 in the RAW264.7 cell line. **b**. Effects of CaMKIV-siRNA on the expression of pCaMKIV, CaMKIV and HMGB1 in the RAW264.7 cell line. The results were selected from triplicates of independent experiments

Whether pCaMKIV can regulate the expression of HMGB1 is an important topic in this research. For further investigation, KN93 was used for incubation with RAW264.7 cell line at different concentrations (Fig. 3a). In particular, pCaMKIV significantly decreased with the increase in KN93 concentration. HMGB1 expression also decreased with pCaMKIV. The specific CaMKIV siRNA was also used in this experiment. Fig. 3b shows the siRNA-CaMKIV can decrease the expression of CaMKIV, and pCaMKIV was similarly reduced. These results suggest that the siRNA of CaMKIV functioned well. The expression of HMGB1 was detected by Western blot and decreased significantly compared with the control (Fig. 3b). These results imply that pCaMKIV can regulate the expression of HMGB1.

### pCaMKIV is localized in the nuclei of DRG neurons

**Fig. 4:**
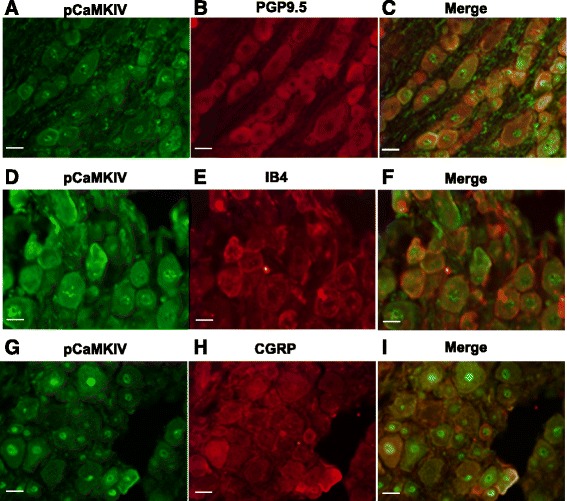
Distribution of pCaMKIV immunereactivity in different subpopulations of DRG neurons at 30 days after STZ treatment. **a** to **i**. Double-immunofluorescence staining shows the distribution of pCaMKIV immunereactivity in different subsets (PGP9.5, IB4, CGRP) of DRG neurons in rats. The images were 400 times magnification and the scale bar was 20 μm

To further investigate the mechanisms of pCaMKIV and the expression of pCaMKIV in DRG neurons, immunohistochemical (IHC) analysis was perfomed to confirm pCaMKIV localization. Neuronal markers PGP9.5, IB4, and CGRP were investigated by IHC in this research. Fig. 4a to C show the colocalization of PGP9.5 and pCaMKIV, whereas Fig. 4d to f and g to I display the colocalization of IB4 and pCaMKIV and that of CGRP and pCaMKIV, respectively. These results suggest that pCaMKIV may be an important regulator in neurons.

### Effect of intrathecal administration of KN93 on diabetic models

**Fig. 5:**
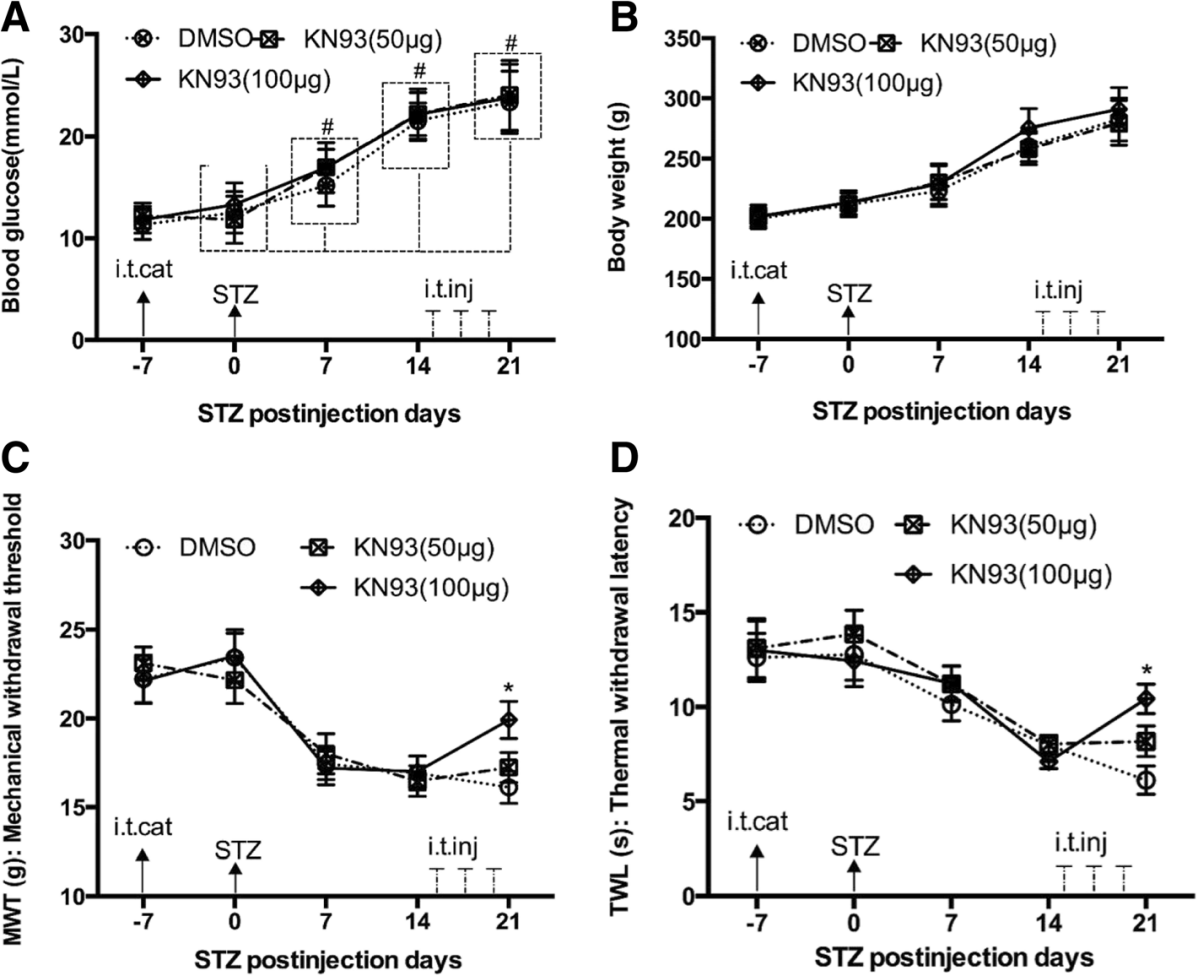
Effects of intrathecal injection of KN93 on blood glucose levels, body weights, mechanical allodynia and thermal hyperalgesia of STZ-induced diabetic rats. **a**. Changes in blood glucose levels after KN93 treatment. **b**. Effect of body weight after KN93 treatment. **c**. Effect of KN93 (100 μg) on STZ-induced mechanical allodynia. **d**. Effect of KN93 (100 μg) on STZ-induced thermal hyperalgesia. Data are presented as means ± SD. ^#^ *P < 0.05* in comparison to the 0 day time point, *n* = 6 per group.*P < 0.05 in comparison with the DMSO group at the same time points, *n* = 6 per group. (i.t.cat: intrathecal catheterization; i.t.inj: intrathecal injection; STZ: streptozotocin intraperitoneal injection)

KN93, a pCaMKIV inhibitor [11], was used to investigate the function of pCaMKIV in diabetic models. KN93 was injected intrathecally at 15, 17 and 19 days after STZ administration; however, the blood glucose and weight gain did not change significantly following STZ administration. Pretreatment with KN93 inhibited STZ-induced mechanical allodynia and thermal hyperalgesia in a time-dependent manner after STZ administration (*P* < 0.05) (Fig. 5).

**Fig. 6:**
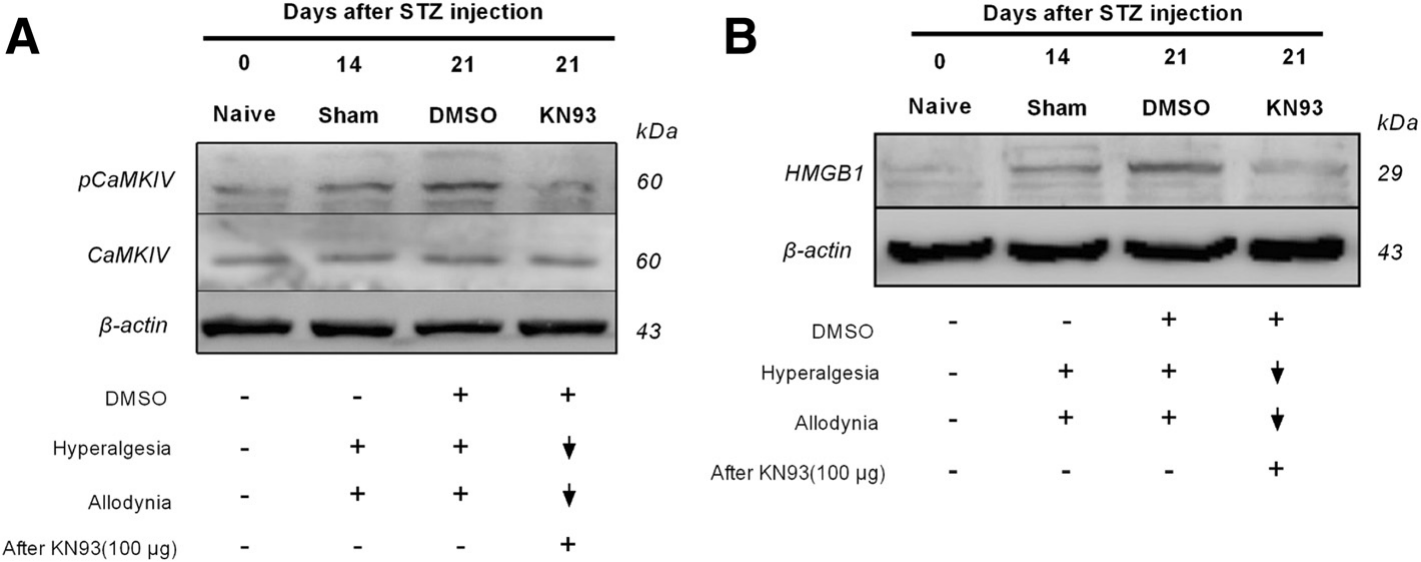
Effects of intrathecal injection of KN93 on the expression levels of pCaMKIV and HMGB1 in DRGs from STZ-induced diabetic rats. **a**. Expression of pCaMKIV after KN93 treatment. **b**. Expression of HMGB1 after KN93 treatment. The results were selected from triplicates of independent experiments

To further investigate the relationship among pCaMKIV, CaMKIV and HMGB1, Western blot was used to determine the change in these proteins (Fig. 6). The expression of pCaMKIV and HMGB1 was shown to increase in DRG after STZ injection. After the intrathecal administration of KN93 (100 μg), the expression of pCaMKIV in DRG decreased significantly following STZ injection. The HMGB1 expression levels also decreased after KN93 treatment.

## Discussion

In the current study, the i.p. injection of STZ induced diabetic neuropathic pain model in rats, as well as increased CaMKIV phosphorylation and HMGB1 expression levels in DRG neurons. In addition, pCaMKIV can regulate the expression of HMGB1. When KN93, a CaMKIV inhibitor, was used in neuropathic pain models, STZ-induced mechanical allodynia and thermal hyperalgesia were inhibited. CaMKIV phosphorylation and HMGB1 expression levels also significantly decreased. Previous reports have indicated that CaMKIV may be a regulator of HMGB1 [11, 17], but the specific mechanisms remain unclear. These results will provide evidence regarding the relationship between CaMKIV and HMGB1.

The present study is the first to demonstrate that pCaMKIV is involved in STZ-induced neuropathic pain in rats. CaMKIV exists in the nuclei of cells and is associated with several transcription factors, such as cyclic-AMP response element-binding protein, AP-1, myocyte enhancer factor 2A, and retinoid orphan receptor family members, which perform pivotal functions in immune response and inflammation [18]. Jackson and Damaj found CaMKIV involvement in both spinal and supraspinal mechanisms of nicotine-induced antinociception; their results suggest that supraspinal nicotine-induced pain mechanisms involve CaMKIV to a larger extent than CaMKII [19]. The current research demonstrated that pCaMKIV in DRG neurons increased in the STZ-induced diabetic neuropathic pain model. This finding suggests that the phosphorylation of CaMKIV is an important regulator in neurons.

The present results also indicated that HMGB1 expression increased in the DRG of STZ-induced type 1 diabetes, rat model, and this finding is consistent with previous reports [20, 21]. However, the mechanism of HMGB1 release in STZ-induced diabetic neuropathic pain model remains unknown. Shibasaki et al. showed that HMGB1 expression increased in the peripheral nerves in response to nerve injury and suggested that this protein contributes to the development of pain hypersensitivity, as revealed by anti-HMGB1 antibody treatment in the neuropathic pain model [20]. In addition, IHC studies demonstrated that HMGB1 levels are upregulated in satellite cells and neurons of the DRG. These results provided a basis to identify the mechanism of HMGB1 in type 1 diabetes.

HMGB1 release is an active process, by which HMGB1 is shuttled from the nucleus to the cytoplasm and then out of DRG neurons. The current results demonstrate that intrathecal administration of KN93, attenuates STZ-induced diabetic neuropathic pain, CaMKIV phosphorylation level, and HMGB1 expression level in DRG. Recently, serine phosphorylation of HMGB1 has been demonstrated to be essential for this translocation event, although the kinase responsible for this mechanism has yet to be identified [22]. Zhang et al. demonstrated CaMKIV-mediated LPS-induced HMGB1 production by translocating the HMGB1 to the nucleus in macrophages [11]. These results indicate different mechanisms underlying the regulation of HMGB1 by CaMKIV. Overall, the data display a mechanism involving the relationship between HMGB1 and CaMKIV in neurons.

## Conclusion

Intrathecal administration of KN93, a CaMKIV inhibitor, can reverse thermal hyperalgesia and mechanical allodynia in STZ rats. These effects may be partially attributed to the decreased expression of HMGB1 in DRG.

## Abbreviations

STZ: streptozotocin
DRG: dorsal root ganglion
HMGB1: high-mobility group box 1
pCaMKIV: phospho-calmodulin-dependent-protein-kinase IV
WB: Western blot
CaMKs: Calmodulin-dependent protein kinases
IHC: immunehistochemical
MWT: mechanical withdrawal threshold
TWL: thermal withdrawal latency
IB4: isolectin B4
PGP9.5: protein gene product 9.5
CGRP: calcitonin gene-related peptide
N.C. siRNA: negative control siRNA
NS: Normal Saline
